# Thermo-Responsive Poly(*N*-Isopropylacrylamide)-Cellulose Nanocrystals Hybrid Hydrogels for Wound Dressing

**DOI:** 10.3390/polym9040119

**Published:** 2017-03-24

**Authors:** Katarzyna Zubik, Pratyawadee Singhsa, Yinan Wang, Hathaikarn Manuspiya, Ravin Narain

**Affiliations:** 1 Department of Chemical and Materials Engineering, Donadeo Innovation Centre in Engineering, 116 Street and 85 Avenue, Edmonton, AB T6G 2G6, Canada; kzubik@ualberta.ca (K.Z.); singhsapt@gmail.com (P.S.); yinan1@ualberta.ca (Y.W.); 2 The Petroleum and Petrochemical College, Center of Excellence on Petrochemical and Materials Technology, Chulalongkorn University, Soi Chulalongkorn 12, Pathumwan, Bangkok 10330, Thailand; hathaikarn.m@chula.ac.th

**Keywords:** poly(*N*-isopropylacrylamide), cellulose nanocrystals, hybrid hydrogels, thermal responsive, wound dressing

## Abstract

Thermo-responsive hydrogels containing poly(*N*-isopropylacrylamide) (PNIPAAm), reinforced both with covalent and non-covalent interactions with cellulose nanocrystals (CNC), were synthesized via free-radical polymerization in the absence of any additional cross-linkers. The properties of PNIPAAm-CNC hybrid hydrogels were dependent on the amounts of incorporated CNC. The thermal stability of the hydrogels decreased with increasing CNC content. The rheological measurement indicated that the elastic and viscous moduli of hydrogels increased with the higher amounts of CNC addition, representing stronger mechanical properties of the hydrogels. Moreover, the hydrogel injection also supported the hypothesis that CNC reinforced the hydrogels; the increased CNC content exhibited higher structural integrity upon injection. The PNIPAAm-CNC hybrid hydrogels exhibited clear thermo-responsive behavior; the volume phase transition temperature (VPTT) was in the range of 36 to 39 °C, which is close to normal human body temperature. For wound dressing purposes, metronidazole, an antibiotic and antiprotozoal often used for skin infections, was used as a target drug to study drug-loading and the release properties of the hydrogels. The hydrogels showed a good drug-loading capacity at room temperature and a burst drug release, which was followed by slow and sustained release at 37 °C. These results suggested that newly developed drugs containing injectable hydrogels are promising materials for wound dressing.

## 1. Introduction

Hydrogels are soft and viscoelastic polymeric materials that can retain large amounts of water or biological fluids in their three-dimensional network structure [1,2,3]. These materials can also be designed to undergo reversible volume phase transition in response to environmental changes or external stimuli [4,5]. Due to these unique properties, together with the ability to simulate many physical properties of living tissues and the potential to be biocompatible, hydrogels have been of great interest for various biomedical applications such as drug delivery systems, contact lenses, wound dressing, scaffolds for tissue engineering, and cell encapsulation [6,7,8,9]. However, in some applications, particularly in wound dressing and tissue engineering, excellent mechanical properties are crucial; therefore, the inherent mechanical weakness and brittleness of many hydrogels remain the major drawbacks that limit their use in such applications [7,10,11,12].

There are several approaches that have been employed in attempts to improve the mechanical properties of hydrogels, including double network (DN) hydrogels [13,14], click hydrogels [15], topological (TP) hydrogels [16], macromolecular microsphere composite (MMC) hydrogels [17], and nanocomposite gels [18,19,20,21,22]. Among them, hydrogels with nano-sized particles have received considerable attention [23,24]. As a result, various types of nanoparticles such as hydroxyapatite (nHA) [25], silica [26,27,28], carbon nanotubes (CNTs) [29], and graphene oxide sheets [30] have been demonstrated to be promising in developing hydrogels with improved mechanical properties and unique microstructures. The major issue associated with the use of these nanoparticles in vivo is their potential toxicity [24,31].

Cellulose nanocrystals (CNCs) are attractive materials for potential in vivo application because of their biocompatibility, biodegradability, and relatively low cytotoxicity [31,32,33,34,35]. CNCs, with dimensions ranging from 3 to 30 nm in cross-section and from 100 to 300 nm in length, are highly rod-like crystalline components in cellulose isolated through acid hydrolysis from the amorphous regions of cellulosic plant materials [36,37,38]. They have recently gained attention not only due to their wide availability from sustainable resources but also due to their outstanding mechanical properties [34]. In fact, the elastic modulus of native cellulose crystals, determined using several techniques, yield values up to 150 GPa [39,40]. Moreover, CNCs have mechanical strength in the range of several GPa, which is higher than that of some metals such as steel or alloy, high aspect ratios (>300), and a surface area of hundreds of square meters per gram [41]. Owing to these characteristics, many research groups have applied CNCs as reinforcing agents in polymer hydrogels based on polyvinyl alcohol [42], poly(acrylic acid) [2], poly(ethylene glycol) [3], polyacrylamide [43], and poly(2-hydroxyethyl methacrylate) [44]. In all of these hydrogels, improved stability and enhanced mechanical properties were observed even at low CNC concentration.

Metronidazole (MZ) is a synthetic antibiotic and belongs to the 5-nitroimidazole group, which is highly effective against anaerobic bacteria and protozoa. It has been used systemically and/or topically to treat wound infections, for example, fungating carcinomas and necrotic pressure ulcers [45,46], because of its ability to reduce the malodor of anaerobically colonized wounds (malodorous wounds). Moreover, two studies suggested that topical metronidazole increased epithelialization during wound healing by secondary intention in rats [47,48]. However, most treatments use conventional gel preparations for MZ, which require generous application and need a secondary dressing. Cavity dressings may need to be smeared with gel to aid application [49] which is not convenient to use. Hence, the incorporation of MZ into hydrogels, which can be injected directly to deep wounds or narrow-opening wounds, brings an interesting approach to increase the effectiveness of wound dressings. 

The present work aims to create a simple way to construct thermo-responsive poly(*N*-isopropylacrylamide) (PNIPAAm) hydrogels by incorporating of CNCs to the gel network. PNIPAAm, which exhibits a phase transition near the physiological temperature, has been shown to have versatile applications in the biomedical field as drug or gene delivery carriers, tissue engineering scaffolds, and platforms for biosensing [50,51]. Recently, Cha et al. [52] prepared PNIPAAm-based hydrogels with surface-carboxylated cellulose nanocrystals that were physically dispersed within the gel matrix. Hebeish et al. [53] used NIPAAm monomers along with cellulose nanowhiskers (CNWs) to develop PNIPAAm/CNWs semi-interpenetrating polymeric network hydrogels. However, these hydrogels were synthesized in the presence of crosslinking agents, and only the hydrogel’s structure, morphology, thermal sensitive property, and swelling behavior were examined. Therefore, the goal of this study was to graft *N*-isopropylacrylamide onto the surface of CNCs and polymerize it via free-radical polymerization to form injectable hydrogels without the addition of cross-linkers. The impact of different CNC concentrations on the thermal stability, phase volume transition temperature, swelling ratio, viscoelastic properties, and injectability of the resulting hydrogels was investigated. An attempt was made to achieve thermo-responsive CNC-reinforced injectable hydrogels that can be used as wound dressings, in which increased stability and enhanced mechanical properties are crucial. In order to evaluate the performance of these hydrogels as drug delivery system*,* in vitro drug loading and release studies were performed by using MZ as a model drug.

## 2. Materials and Methods

### 2.1. Materials

The monomer *N*-isopropylacrylamide (NIPAAm) was purchased from Sigma-Aldrich Chemicals (Oakville, ON, Canada) and purified by recrystallization in a benzene/*N*-hexane mixture prior to use. The cellulose nanocrystals (CNC) were provided by Alberta Innovates-Technology Futures (Edmonton, AB, Canada) and used as received. Ammonium persulfate (APS) was obtained from Sigma-Aldrich (St. Louis, MO, USA), and 1,2-*di*-(dimethylamino)ethane (TEMED) was purchased from Omnipur (Gibbstown, NJ, USA). Both reagents were used without further purification. Metronidazole was purchased from Sigma-Aldrich (St. Louis, MO, USA). The pH 7.4 phosphate-buffered saline (PBS) solution was prepared and then adjusted to an ionic strength of 0.1 M with NaCl solution. Milli-Q water (Millipore, Billerica, MA, USA) was used for all the experiments.

### 2.2. PNIPAAm-CNC Hydrogel Preparation

PNIPAAm-CNC hybrid hydrogels were synthesized by free-radical polymerization. Typically, 5 mmol (565 mg) of NIPAAm was dissolved in 4 mL of the milli-Q water with varied concentrations of CNC (1, 5, 10, 20, and 50 mg/mL of water) by sonication for 10 min, followed by magnetic stirring at room temperature. Once the homogenous solution with well dispersed, the CNCs were obtained and cooled in an ice bath, a 1 mL aqueous solution containing 5 mg of APS, and 20 µL of TEMED was added. The solution was then mixed for an additional 30 min under nitrogen and then left for 24 h at room temperature. The control PNIPAAm polymer was synthesized by the same method with no CNC addition. Furthermore, the PNIPAAm-CNC blended hydrogel was also prepared by polymerizing the PNIPAAm polymer first and then adding the sonicated suspension of CNC in a concentration of 50 mg/mL of water into the polymer solution, after which the mixture was mixed for 30 min. Thereafter, the hydrogels were purified by heating the samples to a temperature above the LCST and subsequently washed thoroughly with milli-Q water in order to remove unreacted monomers, and CNCs from the hydrogels. In addition, before and after purification, the dry weights of freeze-dried hydrogels were determined.

### 2.3. Characterizations

#### 2.3.1. Thermogravimetric Analysis (TGA)

The hydrogel thermal properties were characterized by TGA (SDT Q600 TGA/DSC system, TA Instruments, New Castle, DE, USA). To do this, the purified hydrogels were freeze-dried for three days to completely remove any water and then heated under a compressed air purge from 20 to 800 °C at a heating rate of 10 °C/min.

#### 2.3.2. Fourier-Transform Infrared Spectroscopy (FT-IR)

FT-IR spectra of the freeze-dried hydrogel samples were obtained using a Frontier MIR/FIR spectrometer (Perkin Elmer, Waltham, MA, USA) in ATR mode. All spectra were taken between 4000 and 400 cm^−1^ with 32 scans at a resolution of 4 cm^−1^.

#### 2.3.3. Rheology Test

Dynamic frequency sweeps were used for the examination of the viscoelastic properties of hydrogels by using an AR-G2 rheometer (TA Instrument, New Castle, DE, USA) with a 20-mm parallel-plate configuration and with a gap of 53 µm. A solvent trap was used to prevent drying effects. The experiments were conducted at a constant strain (γ) = 1% over an oscillating frequency (ω) range of 0.1–100 rad/s at 20 and 37 °C. All the samples were purified prior to rheology measurements.

#### 2.3.4. Hydrogel Injection

All injectable hydrogels were prepared by dissolving 60 mg of freeze-dried samples in 4 mL of milli-Q water and allowing full swelling at room temperature for 24 h. The hydrogels were subsequently injected through 25G needles into a water bath at 37 °C. The appearances of the hydrogels before and after injection were observed and photographed by a digital camera.

#### 2.3.5. Volume Phase Transition Temperature (VPTT)

The volume phase transition temperature (VPTT) of the thermo-responsive hydrogels was determined by optical absorption spectroscopy (TECAN GENios Pro, Mainz, Germany), as described previously [54]. Briefly, the hydrogels (200 µL in volume, *n* = 3) were loaded into 96-well plates, and an average absorbance was measured at different temperatures, ranging from 20 to 42 °C, with a 1 °C temperature increase every 15 min. VPTT was defined as the temperature at which 50% of the maximum absorbance change was observed.

#### 2.3.6. Equilibrium-Swelling Ratio (ESR)

The pre-weighed dried hydrogels (*W*_d_) were immersed in 10 mL deionized water at a certain temperature until swelling equilibrium was attained. Each gel was then removed from the water bath, tapped with filter paper to remove any excess surface water, and weighed to obtain the swelling equilibrium weight (*W*_e_). The equilibrium-swelling ratio (ESR, g g^−1^) in H_2_O was calculated from the following equation:

(1) $$\mathrm{ESR}=\;\frac{W_{e}-W_{d}}{W_{d}}$$

where *W*_e_ is the weight of the gel at a certain temperature and *W*_d_ is the dry weight of the gel. The study of the ESRs of thermo-responsive hydrogels at different temperatures was carried out in the temperature range of 20–50 °C. All experiments were performed in triplicate, and an average value of three measurements was recorded.

#### 2.3.7. Drug Loading and Release

Metronidazole (MZ) was selected as the model drug in the drug release study. MZ was dissolved in a 0.1 M, pH 7.4 PBS solution to prepare the MZ solutions with two different concentrations of 1 and 5 mg/mL. The 500 mg of freeze-dried hydrogel samples were loaded with MZ by soaking them in 10 mL of each concentration of the MZ solution in the dark at 20 °C for three days. Thus, MZ was loaded into the hydrogels up to equilibrium by swelling in the MZ-containing solution, and then the MZ-loaded hydrogels were used for the in vitro drug release study. The concentration of unloaded MZ in the residual solution was measured with a V-630 UV-vis spectrophotometer (JASCO, Tokyo, Japan) at 320 nm to calculate the percent entrapment of the drug in the hydrogels.

In vitro release studies of the drug at 37 °C were carried out by transferring 500 mg of MZ-loaded hydrogels to dialysis bags (MWCO: 7000 Da) and then placing these in closed glass bottles filled with 10 mL PBS (0.1 M, pH 7.4), stirring at 100 rpm/min. At a predetermined interval, 2 mL aliquots of the environmental buffer solution were removed from the bottles and replaced with 2 mL fresh preheated buffer solution. The concentration of released MZ in the removed PBS was determined using the same UV-vis spectrometry method against the established calibration curve. All release studies were carried out in triplicate, and an average value of three measurements was recorded. Then, the percentages of the cumulative drug release of MZ were calculated as a function of time.

(2) $$\mathrm{Cumulative}\;\mathrm{drug}\;\mathrm{release}\left(\%\right)=\frac{\left(V_{e}\sum_{i=1}^{n-1}C_{i}\;+\;V_{0}C_{n}\right)}{m_{\infty }}\;\times 100\%,$$

where *V*_e_ is the amount of release media taken out each time (2 mL), *V*_0_ is the amount of release media in the glass bottle, *C_i_* is the concentration of MZ released from the hydrogels at a displacement time of *i*, *C_n_* is the concentration of MZ in the sample, and *m*_∞_ is the estimated amount of MZ loaded in the hydrogels and was calculated from the weight difference between the initial MZ solution concentration (before loading) and the remaining MZ solution concentration after loading (*m*_∞_ (mg) = 5 − *m*_residual solution_). 

## 3. Results and Discussion

### 3.1. Synthesis of PNIPAAm-CNC Hydrogels

**Scheme 1:**
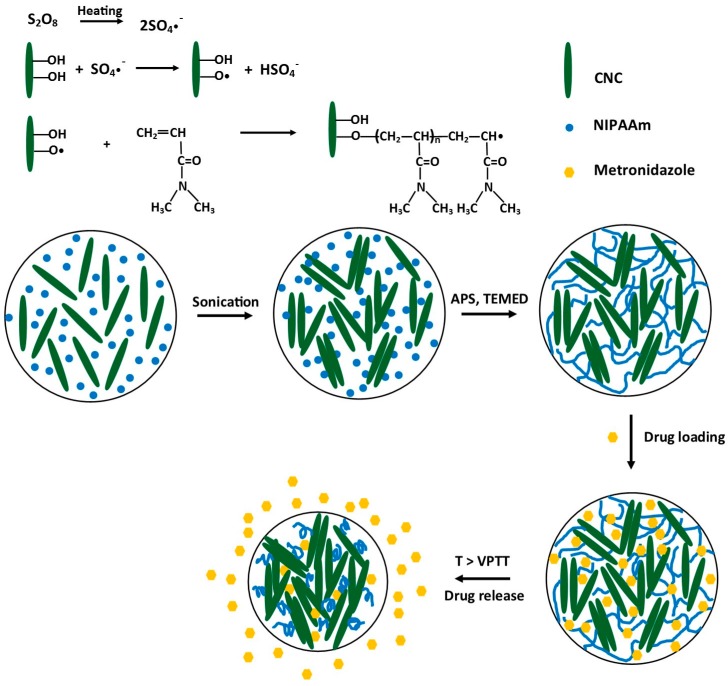
The synthesis process and a schematic representing the structural model, drug loading, and drug release of thermo-responsive poly(*N*-isopropylacrylamide) (PNIPAAm)-cellulose nanocrystals (CNC) hybrid hydrogels.

PNIPAAm-CNC hybrid hydrogels were synthesized by free-radical polymerization, which involves only the mixing of all the components before initiating network formation. As shown in Scheme 1, the polymerization was initiated by adding water-soluble initiator APS and accelerator TEMED, without any additional chemical cross-linkers. The initial sulfate anion radicals produced from the thermal decomposition of the initiator captured hydrogen from the hydroxyl groups on the CNC macromolecules to generate the alkoxyl radicals, which then interacted with the NIPAAm monomers to form the graft copolymer. The resulting hydrogels were denoted as NC-*x* hydrogels, in which *x* corresponds to the varied amounts of CNC; NC-1, NC-5, NC-10, NC-20, and NC-50 represent 1, 5, 10, 20, and 50 mg CNC/1 mL of water used in the hydrogel formation. For comparison, PNIPAAm, consisting of an uncross-linked polymer network immersed in water, and PNIPAAm-CNC blended hydrogels with the highest CNC content were also prepared and denoted as NC-0 and NC-50+, respectively. 

**Figure 1:**
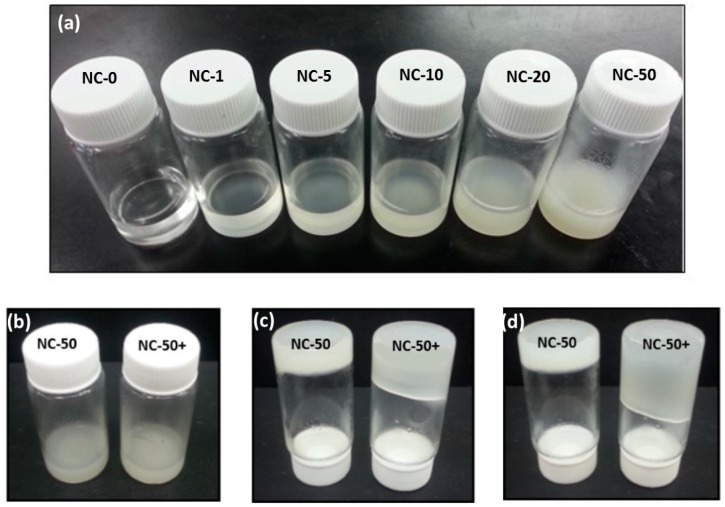
(**a**) Appearance of PNIPAAm-CNC hydrogels with increasing CNC concentration and (**b**) the difference in appearance and flowing between grafted (NC-50) and blended (NC-50+) CNC samples before the flowing test; (**c**) 30 s after inverting the vials; (**d**) 1 min after inverting the vials.

Figure 1a shows the impact of CNC concentration on the visual appearance of the hydrogels. The hydrogels started to turn opaque with the increase in the CNC content in the networks, which may be due to the light scattering at the CNCs’ crystalline region [55]. In addition, all CNC hydrogels exhibited good uniformity with no aggregated CNCs or distinct heterogeneous macroscopic phase separation observed. The hydrogels NC-10, NC-20, and NC-50 appeared in gel structures, which indicated the gelation of CNC suspensions. The increase of CNC concentrations can cause a decrease in the electrostatic double layer distance between CNCs, resulting in the gelation of CNC through physical crosslinking caused by the hydrogen bonds of hydroxyl groups on CNCs [56,57,58]. Moreover, sonication of CNC suspensions can affect the gelation [59].

Figure 1b–d show a comparison of the appearance and flowing ability of CNC grafted (NC-50) and blended (NC-50+) hydrogel samples. Both hydrogels were essentially identical in color (Figure 1b), while NC-50 was more resistant to flow in an inverted vial as compared with the blended hydrogel (Figure 1c,d). This observation suggested a stronger shear stress resistance of NC-50 as NIPAAm was adsorbed and polymerized from the CNC surface and eventually formed covalently and physically CNC-reinforced hydrogel networks to strengthen the mechanical properties of the hydrogel [53,55].

**Figure 2:**
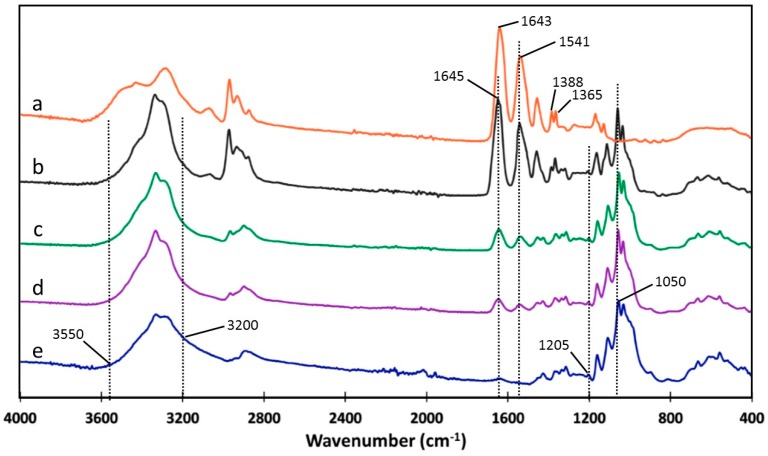
Fourier-Transform Infrared Spectroscopy (FT-IR) spectra of (**a**) NC-0, PNIPAAm; (**b**) NC-50; (**c**) NC-20; (**d**) NC-10; (**e**) CNC.

The FT-IR spectra of PNIPAAm homopolymer (NC-0), PNIPAAm-CNC hydrogels (NC-10, NC-20, and NC-50), and CNC were shown in Figure 2. PNIPAAm had a wide absorption band at 3600–3000 cm^−1^, attributed to NH stretching vibrations, and peaks at 1643 and 1541 cm^−1^, attributed to C=O stretching vibration and N-H deformation vibration in the amide groups, respectively. Two bands at 1388 and 1365 cm^−1^ indicated the stretch vibration of methyl groups (CH(CH_3_)_2_) of PNIPAAm. For CNC characteristics, the strong and broad absorption band at 3550–3200 cm^−1^ showed the alcohol O–H stretching vibrations (intermolecular bond), and several strong peaks in the ranges of 1205–1050 cm^−1^ indicated the C–O stretch absorption of primary, secondary, and tertiary alcohols. In PNIPAAm-CNC hydrogels, the characteristic absorption peaks of CNC in the ranges 3550–3200 cm^−1^ and 1205–1050 cm^−1^ and those of PNIPAAm at 1645 and 1541 cm^−1^ were all identified. Therefore, it was demonstrated that PNIPAAm-CNC hydrogels had the characteristic features of both CNC and PNIPAAm. Interestingly, when normalizing the FTIR spectra to the C–O stretch absorption peak of CNC at 1050 cm^−1^ and then comparing each PNIPAAm-CNC hydrogel sample, the intensities of the amide absorption bands (at 1645 and 1541 cm^−1^) increased with the higher amounts of CNCs being added. Moreover, it could be confirmed by the increase of the alkyl C–H stretching absorption peak of the PNIPAAm at 2965 cm^−1^ [60]. These results indicated that the CNC and PNIPAAm presented in the hydrogels were solely in a crosslinked polymer network after free CNCs and PNIPAAm had been removed from the hydrogel systems in the hydrogel purification process. This statement was supported by the percentages of the dry weight of the purified hydrogels, which were calculated by comparing the dry weights of freeze-dried hydrogels before and after purification. The percentages of the dry weight of NC-1, NC-2, NC-5, NC-20, and NC-50 were 3%, 10%, 22%, 43%, and 91%, respectively. This showed that the increase of added CNC contents led to higher amounts of hydrogel remaining after purification as a result of the formation of crosslinked polymer networks. Owing to the increase of presented CNC being able to hold more amounts of PNIPAAm within the networks, higher amounts of PNIPAAm presented in the hydrogels. Additionally, the intense absorption of the carbonyl stretching vibration (amide I) of PNIPAAm-CNC hydrogels shifted to 1645 from 1643 cm^−1^ for PNIPAAm, which suggested that there were inter-and/or intra-molecular interactions existing in between the PNIPAAm and CNCs, likely through hydrogen bonding or van der Waals force [61]. Thus, the FT-IR results confirmed that the chemically and physically crosslinked PNIPAAm-CNC hydrogels were successfully synthesized [61,62]. 

### 3.2. Thermogravimetric Analysis

**Figure 3:**
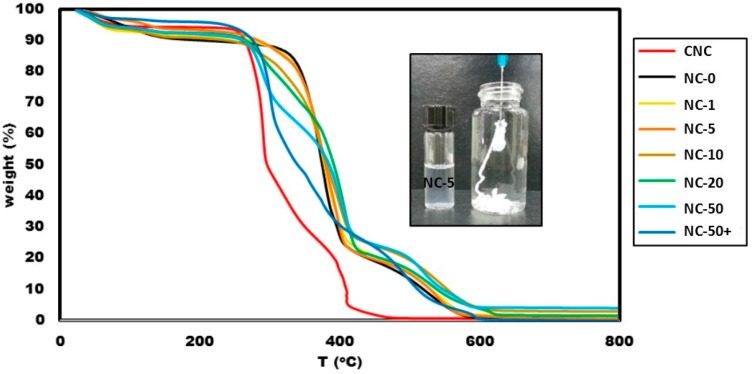
TGA thermograms showing thermal degradation behaviors of a pure CNC sample and prepared PNIPAAm-CNC hybrid hydrogels with various amounts of CNC content.

The hydrogels thermal stability was evaluated by TGA, and the results were shown in Figure 3. The weight of all the studied samples decreased progressively as the temperature increased. The initial weight loss for all hydrogel samples was attributed to the evaporation of residual moisture in the dried hydrogel matrices. PNIPAAm homopolymer was found to be the most thermally stable and started to decompose in a temperature range of 300–580 °C, while CNC was comparatively the least thermally stable, having degradation at 250 to 475 °C (Figure 3). Interestingly, the addition of CNCs to PNIPAAm led to thermal instability. This decrease in the thermal stability of hydrogels could be explained by the high surface area of cellulose nanoparticles, which provided more explosive surface area, leading to the acceleration of their thermal degradation [63], and high amounts of CNCs destroyed the interaction between polymer molecules, resulting in an increase in PNIPAAm molecular mobility [64]. Therefore, the increase of CNC contents resulted in more gradual thermal transitions that occurred within slightly wider temperature ranges. As expected, the blended CNC hydrogel sample (NC-50+) exhibited different thermal behavior to its grafted counterpart (NC-50), showing less gradual thermal transition. Moreover, the degradation temperatures at different stages of decomposition for the blended CNC hydrogel mostly shifted to lower values in comparison to all the grafted hydrogels. 

### 3.3. Volume Phase Transition Temperature (VPTT)

**Figure 4:**
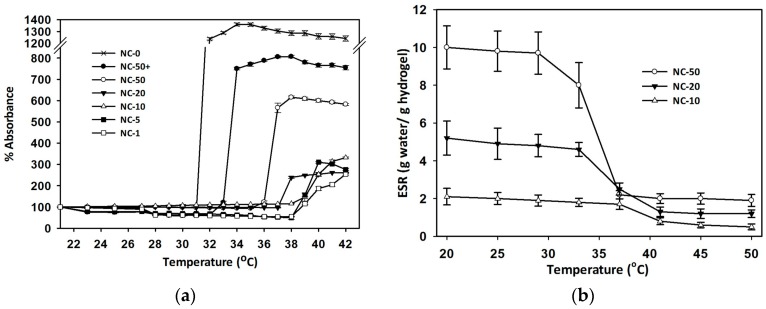
Thermo-responsive properties of PNIPAAm-CNC hybrid hydrogels: (**a**) volume phase transition temperature (VPTT) profile of hydrogels NC-0, NC-50+, NC-50, NC-20, NC-10, NC-5, and NC-1 showing the values of 32, 34, 36.2, 37.5, 38.5, 39, and 39 °C respectively; (**b**) equilibrium swelling ratio (ESR) of PNIPAAm-CNC hydrogels at different temperatures.

The VPTT is a key parameter of thermo-responsive hydrogels. Below the VPTT, hydrogels are hydrophilic and swollen in water. In contrast, above the VPTT, they become hydrophobic and abruptly decrease in volume (collapsed state) [65,66]. The VPTT of PNIPAAm-CNC hydrogels was investigated by optical absorption spectroscopy using a fixed volume of fully swollen hydrogels (200 µL), which were obtained by soaking 20 mg of freeze-dried hydrogels in distilled water for three days. All of the PNIPAAm-CNC hydrogels clearly exhibited phase transition behaviors, as shown in Figure 4a. The VPTT for NC-50+, NC-50, NC-20, NC-10, NC-5, and NC-1 were 34, 36.2, 37.5, 38.5, 39, and 39 °C, respectively, which were higher than a typical low critical solution temperature (LCST) of about 32 °C for PNIPAAm homopolymer in aqueous solution [67]. Generally, the VPTT of thermo-responsive hydrogels is mostly controlled by the relative hydrophobicity of the system [68]. The hydrophobic/hydrophilic balance of PNIPAAm-CNC hydrogels was changed because of the introduction of CNC units into the PNIPAAm network. The presence of hydrophilic CNC decreased the hydrophobicity of the hydrogels, resulting in the increase of VPTT. However, the NC-50 containing the highest amount of CNC showed the lowest VPTT among all the hybrid CNC hydrogels. This could be explained by the fact that, after purification, free CNCs and free PNIPAAm were removed; thus there were only crosslinked networks of PNIPAAm and CNCs left in the hydrogel systems, in which the increasing amounts of presented CNC can hold more amounts of PNIPAAm within the networks [53]. Consequently, the denser PNIPAAm amounts increased the hydrophobicity of the hydrogels; thus the VPTT of NC-50 was closer to that of PNIPAAm homopolymer. In the case of the PNIPAAm-CNC blended hydrogel, the VPTT increased slightly because the PNIPAAm chains could respond to temperature changes quickly, showing that there was no network interaction between PNIPAAm and the CNCs.

### 3.4. Equilibrium Swelling Ratio (ESR)

To evaluate the thermo-sensitive properties of hydrogels, the temperature-dependent swelling ratio is one of the most important parameters required. Figure 4b shows the equilibrium swelling ratios (ESR) of the PNIPAAm-CNC hydrogels at varied temperatures from 20 to 50 °C. The ESR of all hydrogels decreased as the temperature increased according to their thermo-responsive behaviors [69,70]. At low temperatures, these hydrogels absorbed water and became swollen; when the temperature increased, they shrank in volume, resulting from the collapse of the PNIPAAm coils. The ESR values changed drastically at the temperature range of 36 to 41 °C, exhibiting the phase transition behaviors of the hydrogels that agreed with their VPTT results. Obviously, the increase of CNC contents enhanced the ESR of the PNIPAAm-CNC hydrogels; NC-50 had the largest ESR value of 10 g water/g hydrogel. The higher water holding capacities of hydrogels could be attributed to the increased hydrophilicity of the systems as a result of the addition of CNCs, which can form hydrogen bonds easily with water molecules using the hydroxyl groups [71]. Moreover, at the temperature higher than the VPTT, the ESR values of all hydrogels were not zero, indicating that the CNCs were still able to hold some water within the hydrogels; in particular, NC-50 could retain about 2 g water/g hydrogel due to the high content of CNCs incorporated in it.

### 3.5. Rheology

**Figure 5:**
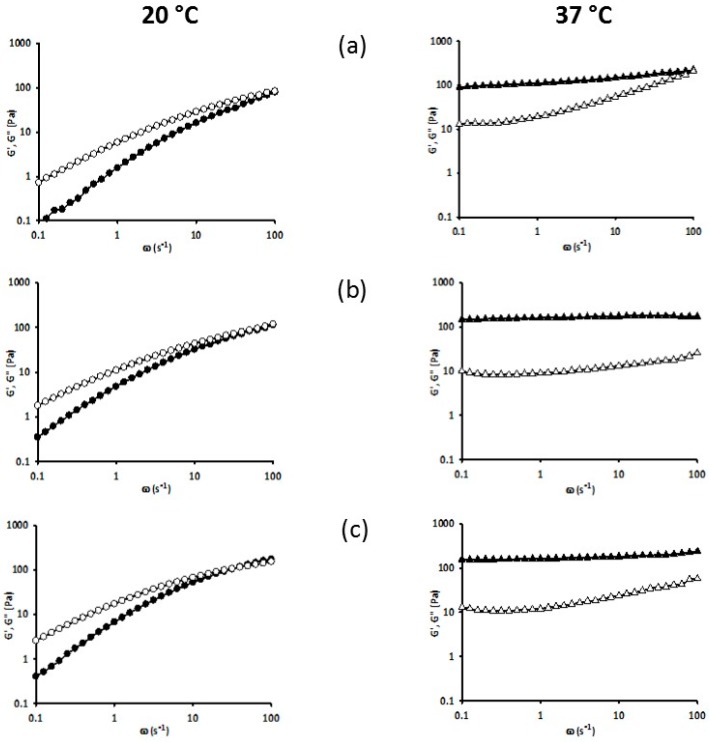

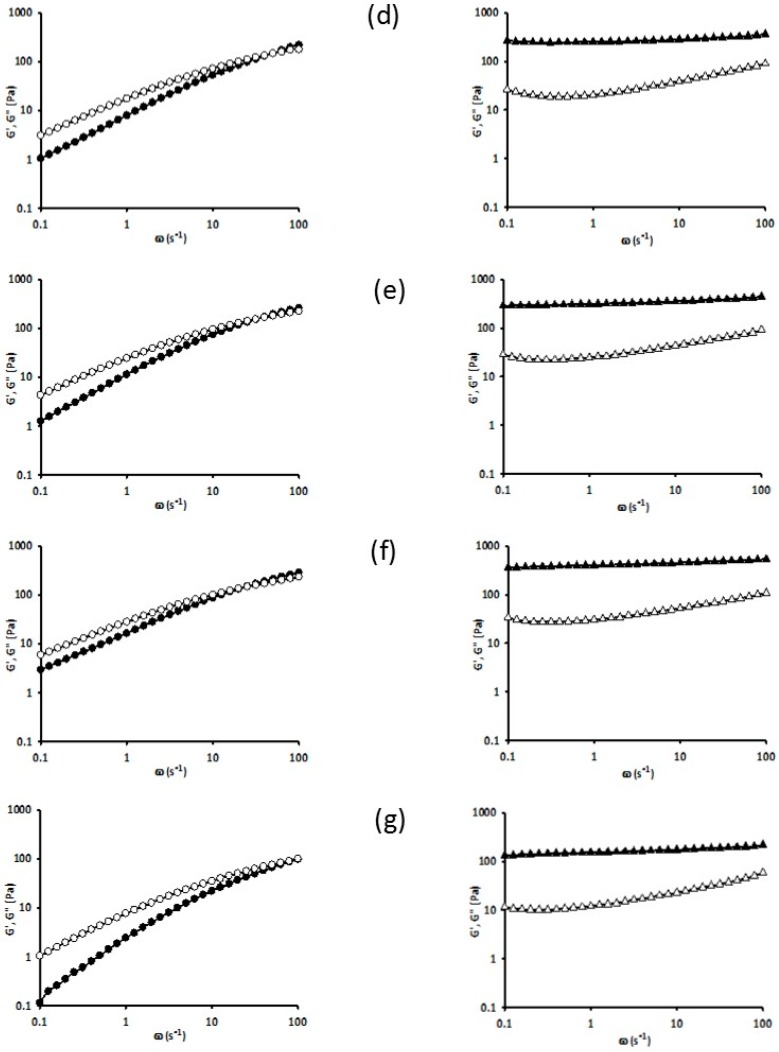
Oscillatory frequency sweeps of shear storage modulus (filled symbols), *G*’, and the shear loss modulus (hollow symbols), *G*”, for PNIPAAm-CNC hybrid hydrogels and blended hydrogel collected at two temperatures: 20 °C (left panels) and 37 °C (right panels) with different CNC concentrations: (**a**) NC-0; (**b**) NC-1; (**c**) NC-5; (**d**) NC-10; (**e**) NC-20; (**f**) NC-50; and (**g**) NC-50+.

The PNIPAAm-CNC hybrid hydrogels were subjected to frequency sweeps at 20 and 37 °C in a rheometer to further investigate the hydrogels’ mechanical properties. The left panels of Figure 5 present the plot of storage (*G*’) and loss moduli (*G*”) versus oscillatory frequency at 20 °C. The results revealed that for all of the hydrogels, both the *G*’ and *G*” values increased monotonically with angular frequency. Simultaneously, the elastic moduli *G*’ were gradually getting closer to the viscous moduli *G*” at high angular frequencies. Interestingly, for the PNIPAAm hydrogel, hydrogels with the lowest CNC content (NC-1), and blended CNC (NC-50+), the crossover frequencies were not observed and the *G*” values were always higher than *G*’ throughout the entire frequency range. These results clearly indicated a liquid-like behavior for those samples. On the other hand, hydrogels with 5, 10, 20, and 50 CNC contents showed a sol-like behavior (*G*’ < *G*”) at low frequencies but exhibited a gel-like property (*G*’ > *G*”) at higher angular frequency ranges [72,73]. It was also observed that the crossover frequency decreased with an increase in CNC concentration in the gel network. Since hydrogels with higher crossover frequencies are always associated with weaker mechanical properties [74], these results also demonstrated that the addition of higher amounts of CNC strengthened the networks of hydrogels. 

The rheology of the hydrogels at 37 °C (Figure 5, right panels) was found to be significantly different from that of the ones at 20 °C. For all the hydrogels, the *G*’ values were higher than the corresponding *G*” values throughout the entire frequency range. This result indicated that, at 37 °C, the hydrogels were predominantly elastic and showed stronger mechanical properties compared to the samples tested at 20 °C [74]. Additionally, the *G*’ curves were relatively independent of frequency throughout the entire range studied, showing typical gel response with little change in viscoelastic characteristics [75,76,77]. These data indicated that PNIPAAm-CNC hybrid hydrogels formed rather stable and strong gel networks at temperatures above the VPTT [72].

It was also observed that the increase of CNC content in hydrogels resulted in higher *G*’ and *G*” values at both temperatures investigated. These results demonstrated a CNC concentration dependence of the PNIPAAm-CNC hydrogels’ rheological properties, as CNCs play important roles as multifunctional cross-links and bridge the neighboring polymer chains [78,79]. However, in the case of CNC blended hydrogel (NC-50+), since nanoparticles were randomly dispersed rather than interacting with polymer chains in gel networks, the *G*’ and *G*” values were even lower compared to the ones measured from the low CNC grafted hydrogels (NC-1 and NC-5). 

### 3.6. Hydrogel Injection

**Figure 6:**
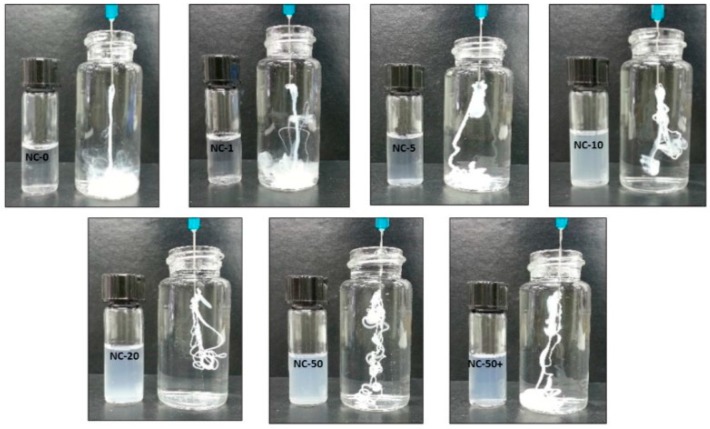
Appearances of the liquefied 15% (*w*/*v*) PNIPAAm-CNC hydrogel samples with different CNC loadings before injection (small glass vials) and their appearance changes during their injection into 37 °C water bath (large glass vials).

To investigate the effect of different CNC concentrations on the hydrogels’ injectability, the re-swollen 15% (*w*/*v*) hydrogels were injected into a water bath at 37 °C (Figure 6). It was noticed that immediately after starting the injection, all solutions began to undergo phase transition and to form gels, which showed a good agreement with the results obtained from the rheology tests (Figure 5b). Interestingly, for hydrogels with low CNC loadings (<10 mg/mL), the gelation was noticeably diminished as the resulting hydrogels were unable to maintain their structure with increasing injection dosage. This instability was manifested by increasing change in the water bath’s turbidity. On the other hand, the samples with high CNC concentration (>10 mg/mL) exhibited higher stability in water and remained coherent for longer during the entire injection period. The explanation could be that the higher concentration of CNCs caused narrower distances between CNC molecules, resulting in the higher chance of the hydrogen-bonding interactions among CNCs for the gelation occurrence when the temperature increased. Moreover, the rod-like structures of the CNCs could act as the bridges to connect the PNIPAAm chains by creating PNIPAAm-CNC hydrogen bonds to support the gel network [79]. This observation implied that CNCs reinforced the hydrogels to enable the gels to maintain their structure upon injection [55]. 

### 3.7. Drug Loading and Release

From the characterization results of PNIPAAm-CNC hybrid hydrogels, the NC-50 hydrogel showed the best suitable properties for use as an injectable wound dressing and had a VPTT of 36.2 °C, the highest ESR value, high mechanical properties at both temperatures of 20 and 37 °C, and the greatest stability in water after injection. Therefore, the NC-50 was selected for further studies. 

**Figure 7:**
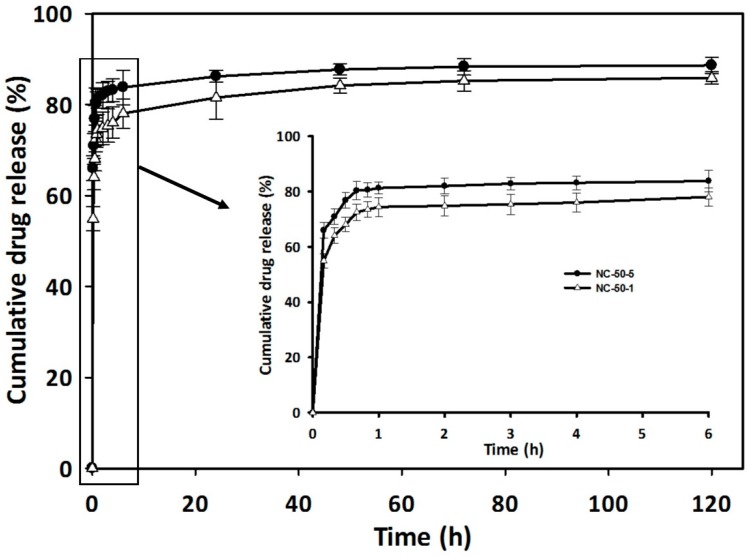
In vitro cumulative drug release of metronidazole (MZ) from NC-50 hydrogels in phosphate-buffered saline (PBS) with pH 7.4 at 37 °C.

In order to evaluate the ability of the PNIPAAm-CNC hybrid hydrogels to effectively deliver MZ, in vitro MZ release from NC-50 hydrogels at 37 °C was studied (Scheme 1). Firstly, MZ was loaded into the hydrogels by incubating the dried hydrogels in two different concentrations of MZ PBS buffer solution (1 mg/mL MZ denoted as NC-50-1 and 5 mg/mL MZ denoted as NC-50-5). The loading contents of MZ were 9.2 and 47.3 mg MZ per 1 g swollen hydrogel for NC-50-1 and NC-50-5, respectively. The increase of MZ loading about five times indicated that the drug loading capacity of the NC-50 hydrogel was affected by the initial drug loading amounts. This could be due to the high water-uptake ability of hydrogels, which could hold the aqueous solution of the drug effectively as long as the drug still remained in its dissolved form. Next, after drug encapsulation, the drug release experiment was carried out at 37 °C above the VPTT. Figure 7 shows the release profiles of MZ from the NC-50 hydrogels as a function of time. The results exhibited that the MZ was released quickly within the first 40 min, showing the burst release at the early stage of the PNIPAAm-CNC hydrogels, which resulted from the PNIPAAm segments collapsing and releasing the drug out of the hydrogel network. At 40 min, the percentage cumulative drug release of the NC-50-5 (80%) was higher than that of NC-50-1 (72%), indicating that the release rates of the NC-50 hydrogels depended on the amounts of the drug present in the matrices. It was in accordance with the drug concentration gradient, for which the higher gradient can be a driving force for the drug release [80]. After the burst release stage, the MZ was sustained, released, and reached the maximum release in 24 h; this was 86% for NC-50-5 and 82% for NC-50-1. The maximum release of MZ from PNIPAAm-CNC hydrogels did not reach 100%, even when running the release study for 120 h. The explanation for unreleased MZ could be that the MZ is bound by the polymer network with weak hydrogen bonds between the polar groups of MZ and hydrophilic groups of the polymers; another explanation is the strong ionic interactions between nitrogen in the molecule’s ring [81,82]. However, the initial burst release of MZ from the NC-50 hydrogels at an early stage could favor the clinical therapeutic effect, especially the antibacterial effect of MZ, which requires certain amounts of the drug to reach the effective concentration quickly after administration to maximize bacteria eradication and minimize the drug resistance of bacteria [83,84,85].

## 4. Conclusions

PNIPAAm-CNC hybrid hydrogels, which combined the stimuli-responsivity of PNIPAAm with the unsurpassed physical properties of CNC, were successfully fabricated without using any additional cross-linkers via a free radical polymerization process. The hydrogel structures were chemical and physical crosslinked networks. The investigation of the PNIPAAm-CNC hybrid hydrogel properties affected by the incorporated CNC demonstrated that the CNCs can reinforce the hydrogels chemically with the covalent crosslinks of their hydroxyl groups with PNIPAAm and physically with their hydrogen bonding ability, which can interact with neighboring CNC and PNIPAAm chains. The obtained PNIPAAm-CNC hybrid hydrogel containing the highest CNC content exhibited the best mechanical property, the optimum VPTT value of 36.2 °C, the highest ESR value, and the greatest stability after injection. Consequently, this hydrogel was studied for the drug loading and in vitro drug release of metronidazole, an antibiotic used in wound infections. The results showed that PNIPAAm-CNC hybrid hydrogels had a good drug-loading capacity at room temperature and a burst drug release followed by a slow and sustained release at 37 °C. Therefore, these PNIPAAm-CNC hybrid hydrogels may have a potential application as an injectable hydrogel for wound dressing. For future work, the antimicrobial effects of the released metronidazole from the hydrogels will be studied to confirm the efficiency of the PNIPAAm-CNC hybrid hydrogels as a wound dressing.
